# RASMA: a reverse search algorithm for mining maximal frequent subgraphs

**DOI:** 10.1186/s13040-021-00250-1

**Published:** 2021-03-16

**Authors:** Saeed Salem, Mohammed Alokshiya, Mohammad Al Hasan

**Affiliations:** 1 North Dakota State University, Fargo, ND, 58102 USA; 2 Indiana University–Purdue University Indianapolis, Indianapolis, IN, 46202 USA

**Keywords:** Biological networks, Subgraph enumeration, Frequent subgraphs, Maximal subgraphs, Reverse search

## Abstract

**Background:**

Given a collection of coexpression networks over a set of genes, identifying subnetworks that appear frequently is an important research problem known as mining frequent subgraphs. Maximal frequent subgraphs are a representative set of frequent subgraphs; A frequent subgraph is maximal if it does not have a super-graph that is frequent. In the bioinformatics discipline, methodologies for mining frequent and/or maximal frequent subgraphs can be used to discover interesting network motifs that elucidate complex interactions among genes, reflected through the edges of the frequent subnetworks. Further study of frequent coexpression subnetworks enhances the discovery of biological modules and biological signatures for gene expression and disease classification.

**Results:**

We propose a reverse search algorithm, called RASMA, for mining frequent and maximal frequent subgraphs in a given collection of graphs. A key innovation in RASMA is a connected subgraph enumerator that uses a reverse-search strategy to enumerate connected subgraphs of an undirected graph. Using this enumeration strategy, RASMA obtains all maximal frequent subgraphs very efficiently. To overcome the computationally prohibitive task of enumerating all frequent subgraphs while mining for the maximal frequent subgraphs, RASMA employs several pruning strategies that substantially improve its overall runtime performance. Experimental results show that on large gene coexpression networks, the proposed algorithm efficiently mines biologically relevant maximal frequent subgraphs.

**Conclusion:**

Extracting recurrent gene coexpression subnetworks from multiple gene expression experiments enables the discovery of functional modules and subnetwork biomarkers. We have proposed a reverse search algorithm for mining maximal frequent subnetworks. Enrichment analysis of the extracted maximal frequent subnetworks reveals that subnetworks that are frequent are highly enriched with known biological ontologies.

## Background

Advances in genome technologies allows for the probing of thousands of genes at the same time through the use of mRNA sequencing and gene expression microarray. Gene expression analysis on such microarray data is then used for discovering gene clusters that have similar expression profiles. Such analysis can also be used for obtaining dysregulated genes that can be used as markers for solving various disease classification tasks.

However, research has revealed that genes do not work in isolation and often a single gene does not have an independent effect on a phenotype, rather multiple genes interact together to control that phenotype. Gene coexpression networks can be used to capture such correlation among genes [1]. Given a gene expression dataset, a coexpression network is built in which the nodes represent genes and a link exists between a pair of genes if the corresponding genes exhibit significant correlation in the microarray analysis [2, 3]. Traditionally gene expression datasets are analyzed independently. However, functional annotation and biological inference based on a single gene coexpression dataset has limitations due to experimental noise [2]. To alleviate experimental noise, multiple gene expression datasets can be analyzed concurrently in a single study. So, recent research has focused on mining biologically interesting gene coexpression subneworks from multiple heterogeneous gene expression datasets.

A set of genes that have similar expression profiles in multiple experiments is more likely to represent a biological module [1, 2]. The integrative analysis of multiple gene expression datasets enables the discovery of significant interactions involved in complex biological processes, and has been employed for functional annotation [1], active module discovery [4], and biomarker discovery [5]. An approach to identify these coexpression subnetworks is to mine significant subgraphs over multiple gene expression networks. Careful study of these significant subgraphs can lead to the identification of functional modules and the discovery of interesting genes interactions that play key roles in complex diseases [6].

Existing algorithms for mining significant subgraphs from coexpression networks mainly follow network clustering [1], approximate and frequent subgraph enumeration approaches [2, 7], or a combination of both. A subgraph that appears in at least a user-defined threshold of the graphs is called a frequent subgraph. A frequent subgraph that is not a subgraph of any larger frequent subgraph is called a maximal frequent subgraph. Mining all frequent and maximal frequent subgraphs is challenging as coexpression networks are generally large, sometimes having tens of thousands vertices. On such large graphs, various algorithmic steps of traditional frequent subgraph mining algorithms [8–10], such as, candidate generation and pruning, graph and subgraph isomorphism are not efficient.

A special class of graphs is the graphs with unique-label nodes, e.g., gene coexpression networks, where no two nodes in the same graph have the same label. For such networks, the computationally-intensive procedures of subgraph and graph isomorphism are not required for mining uniquely-labeled graphs. Moreover, the tasks of candidate generation and pruning is much simpler for uniquely-labeled graphs. The problem of mining frequent subgraphs from graphs with unique vertex labels has received less attention. One of the early algorithms for mining frequent subgraphs from graphs with unique labels is MULE (Mining Uniquely Labeled Edgesets) by Koyuturk et. al. [11]. In the experiments section, we compare our proposed algorithm with MULE.

In this paper, we propose a novel reverse search algorithm for enumerating all edge-induced connected subgraphs of a graph. The reverse search utilizes the shortest distance between edges to check for valid subgraph extensions. Building on this enumeration approach, we propose an algorithm for mining all frequent and maximal frequent subgraphs from a graph database, in which the vertices of each graph has a distinct label. To efficiently mine all maximal frequent subgraphs, we propose two pruning rules that eliminate futile search subtrees in the frequent subgraph enumeration tree. These pruning strategies result in significant improvement in the running time of the algorithm. We demonstrate the effectiveness of the proposed algorithms with the pruning strategies on gene coexpression graphs, and show that the proposed algorithm is orders-of-magnitude faster than existing algorithms.

### Related work

The backbone of frequent subgraph mining algorithms is the enumeration strategy employed for enumerating all connected subgraphs as potentially all connected subgraphs could be frequent. Frequency and feature constraints (e.g., similar node labels) are then enforced while enumerating the subgraphs. In sparse graphs, the number of connected subgraphs is much smaller than the number of all subgraphs. Moreover, the number of subgraphs that satisfy the frequency or feature constraints is much smaller than the number of connected subgraphs.

Koyuturk et. al. [11] proposed the MULE (Mining Uniquely Labeled Edgesets) algorithm for mining frequent subgraphs of a given collection of graphs, $\mathcal {G}$. A subgraph is frequent if the number of graphs it appears in, referred to as support, is at least a user-specified minimum number of graphs. Moreover, an extension to the MULE algorithm was proposed to mine the closed and maximal frequent subgraphs. A closed frequent subgraph is a frequent subgraph that does not have a supergraph with the same supporting graphs. A maximal frequent subgraph does not have any frequent supergraph. At the core of the MULE algorithm is a depth-first enumeration approach based on backtracking for visiting all connected edge-induced subgraphs of a graph. The enumeration approach in the MULE algorithm visits each subgraph in the enumeration tree only once. A subgraph is only extended with edges in the candidate edgeset. The set of candidate edges for a subgraph is defined based on the set of edges visited and the current edges in the subgraph. In the MULE algorithm, at each search node in the search space, the set of subgraphs generated from a given subgraph is not always the set of all supergraphs of a given subgraph because the missing supergraphs would be visited from other subgraphs.

Because the frequency constraint satisfies the downward closure property, the minimum support constraint is enforced while traversing the subgraph lattice and a futile search branch is pruned once an infrequent subgraph is encountered. The downward closure property guarantees that all supergraphs of an infrequent subgraph are infrequent. The number of frequent subgraphs in a graph dataset is very large, especially for small support thresholds. For downstream analysis of these frequent subgraphs, it is often desired to mine a representative set of these frequent subgraphs. A representative set is a subset of the frequent subgraphs such that every frequent subgraph not in the representative set is similar (high overlap) with at least one subgraph in the representative set. Mining a set of representative subgraphs is suitable when it is computationally infeasible to mine all frequent subgraphs. Several approaches have been proposed to mine a succinct set of frequent subgraphs, including maximal frequent and close frequent subgraphs [7]. To highlight the challenges of mining all frequent subgraphs, we run the MULE algorithm on a dataset of 35 graphs, used in the experiments (10,000 nodes, average number of edges 145,114). The MULE algorithm takes hours to generate all frequent subgraphs, depending on the minimum support threshold employed. Moreover, the MULE algorithm generates millions of frequent subgraphs, while the number of maximal subgraphs is in the thousands.

For maximal frequent subgraphs, if a frequent subgraph does not have any frequent supergraph in the enumeration tree, then it is locally maximal frequent. The MULE algorithm checks if the locally maximal frequent subgraph is a subgraph of an already mined maximal frequent subgraph to ensure that the locally maximal frequent subgraph is indeed a maximal frequent subgraph. The set of discovered maximal frequent subgraphs that has to be kept in memory can be very large and thus checking if a subgraph is a subgraph of an already discovered maximal frequent subgraph can be computationally expensive. Another limitation of the MULE algorithm is that it does not have pruning strategies that eliminate the traversal of search branches that would result in locally maximal frequent subgraphs that are not globally maximal frequent subgraphs. For the special case when the graph dataset has a single graph, and minimum support of 1, the MULE algorithm enumerates all frequent subgraphs of the single graph while in fact there is only one maximal frequent subgraph that is the graph itself.

Another approach for enumerating all connected subgraphs was proposed in [12]. The main idea of the approach is that for a given vertex, the set of all connected induced subgraphs can be partitioned into two groups: the subgraphs that have the vertex, and the subgraphs that do not have the vertex. The recursive algorithm has an amortized computation time of *O*(1) for each vertex-induced subgraph. The algorithm in [12] has amortized computation time while our proposed algorithm has a linear delay. The algorithm in [12] can be adapted to solve the edge-induced subgraph enumeration problem, however, it is not clear if the new algorithm will have an amortized computation time of O(1).

Reverse Search is a recent search approach for enumeration problems [13]. The basic idea of reverse search is to arrange all objects to be enumerated in a tree, where each search node has a unique parent node. A major task of a reverse search algorithm is the definition of a *parent* operation on the sets being enumerated that reduces a node in the tree to its unique parent node [14]. All the objects to be enumerated form an enumeration tree with tree nodes representing edges and the connections between objects and the corresponding parent are represented by edges. A *child* operation, defined by inverting the *parent* operation, determines if an object is a valid child of a given parent object. The enumeration tree is constructed by applying a depth-first traversal, starting from a canonical root and employing the *child* operation to generate objects. Several reverse search-based algorithms have been proposed for solving traditional enumeration problems, including all induced connected subgraphs, all spanning trees of a graph, all topological orderings of an acyclic graph, all dense subgraphs of a graph, and all maximal independent sets of a graph [13, 14].

A reverse search algorithm, RS-MST, for enumerating all vertex-induced connected subgraphs has been introduced in [13] where the parent subgraph of a subgraph *G* is obtained by removing the vertex with the minimum degree in the spanning tree of *G*. A subgraph resulting from extending a subgraph *G* with a vertex *v* is a valid child of *G* if vertex *v* is a vertex with the minimum degree in the subgraph formed by adding the vertex *v* to the subgraph *G*. A similar approach can be applied for mining all edge-induced subgraphs. For these two reverse search algorithms, finding the MST to check for valid subgraph extension is a costly operation, considering that some extensions (invalid ones) will not be pursued in constructing the enumeration tree and will not be reported. The delay for the RS-MST algorithm is cubic in the number of nodes since we have to extract the minimum spanning tree for each extension and in the worst case none of the extensions is a valid child. A related problem to the enumeration of all connected induced subgraphs is the problem of enumerating all connected induced subgraphs of size at most *k*. Several algorithms have been proposed for solving this problem [15, 16]. When *k* equals the number of nodes in the graph, the enumeration of all induced subgraphs of size at most *k* and the all connected induced subgraphs enumeration problem become identical. A recent article of the algorithms for mining all connected induced subgraphs of size at most *k* has recently been published [17]. In [18], we proposed a reverse search algorithm for enumerating all vertex-induced connected subgraphs of a graph. The parent operation is based on the shortest distance of the newly added vertex to the first vertex that was added to the subgraph. The algorithm outperformed existing methods for vertex-induced subgraph enumeration. Moreover, we employed the enumeration approach to mine all maximal cohesive subgraphs from vertex-attributed graphs. The proposed method takes an edge-growth approach to mine all connected frequent edgesets.

## Methods

The backbone of the proposed frequent subgraphs mining algorithm is an approach to enumerate all connected edge-induced subgraphs of a single graph. We first explain our enumeration approach for all connected edge-induced subgraphs and then extend this approach to mine all frequent and maximal connected subgraphs.

### Preliminaries

Let *G*=(*V*,*E*) be an undirected graph, where *V*={*v*_1_,⋯,*v*_*n*_} denote the set of vertices and $ E\subseteq \binom {V}{2}$ is the set of edges. For a vertex *v*_*i*_∈*V*, *i* is a unique identifier of that vertex, which is fixed but arbitrarily assigned.

**Subgraph**

A graph *G*_*s*_=(*V*_*s*_,*E*_*s*_) is a *subgraph* of *G*=(*V*,*E*), denoted as *G*_*s*_⊆*G*, if and only if *V*_*S*_⊆*V* and *E*_*s*_⊆*E*.

**Vertex-induced subgraph**

For a graph *G*=(*V*,*E*), and a set of vertices *U*⊆*V*, the vertex-induced subgraph (induced subgraph), denoted as *G*[*U*], is the subgraph *G*[*U*]=(*U*,*E*_*U*_) whose vertexset is *U* and the edgeset *E*_*U*_ includes all edges whose endpoints are in *U*.

**Edge-induced subgraph**

For a graph *G*=(*V*,*E*), and an edgeset *S*⊆*E*, the edge-induced subgraph, denoted as *G*[*S*], is the subgraph *G*[*S*]=(*V*_*S*_,*S*) whose edgeset is *S* and the vertexset *V*_*S*_ includes all the endpoints of edges in *S*.

We call *S*⊆*E* a *connected edgeset* if its corresponding edge-induced subgraph *G*[*S*] is connected. A connected edge-induced subgraph can be uniquely identified by its corresponding connected edgeset and thus the two terms are used interchangeably.

**Edge ordering:**

To maintain an edge ordering, an edge between vertices *v*_*i*_ and *v*_*j*_ is denoted as (*i*,*j*) where *i*<*j*. We define a total order relation on the set of edges in the graph such that (*i*,*j*)≼(*k*,*ℓ*) if *i*<*k* or *i* equals *k* and *j*≤*ℓ*.

The *distance* between two edges, denoted *d*(*e*_*i*_,*e*_*j*_), in a connected graph is the number of non-terminal vertices (connect between edges) in a shortest path between the edges. Using this definition, adjacent edges that share an endpoint have a distance of 1.

**Edge neighborhood:**

For an edge *e*, the set of all adjacent edges of *e* is referred to as the neighborhood of *e*, and is denoted as *N*(*e*). The neighborhood of *e* is defined as the set of edges with a distance of 1 to *e*.

$$N(e) = \left\{e_{i} \in E, d(e_{i},e) =1\right\} $$

**Subgraph neighborhood:**

For an edgeset *U*⊆*E*, the set of neighboring edges in a graph *G*=(*V*,*E*), denoted as *N*(*U*), contains the set of edges not in *U* that have at least one neighboring edge in *U*.

$$N(U) = \left\{e_{i} \in E \setminus U, ~~\exists~e\in U~\textrm{such that} ~d(e_{i},e) = 1\right\} $$

**Anchor edge:**

The smallest edge in an edgeset *U* is denoted *anchor*(*U*), i.e., *anchor*(*U*)=*s* such that *s*≼*e*_*i*_,∀*e*_*i*_∈*U*∖*s*.

**Closer to anchor:**

For a connected edgeset *U*⊆*E* with *s*=*anchor*(*U*), and any two edges *e*_*i*_,*e*_*j*_∈*E*∖*U*, we say that *e*_*i*_ is ‘closer’ to *U* than *e*_*j*_, denoted as (*e*_*i*_≺_*U*_*e*_*j*_), if *d*(*e*_*i*_,*s*)<*d*(*e*_*j*_,*s*) or *d*(*e*_*i*_,*s*)=*d*(*e*_*j*_,*s*) and *e*_*i*_≼*e*_*j*_.

**Utmost edge:**

For a connected edgeset *U* with *s*=*anchor*(*U*), the largest edge in *U* with the longest distance to the anchor edge is called the utmost edge and is denoted as *utmost*(*U*). If there is more than one edge whose distances equals the longest distance, we take the largest edge according to the order relation, i.e., *utmost*(*U*)=*e* such that *e*∈*U*∖*s* and ∀*e*_*i*_∈*U*∖*e* either *d*(*e*_*i*_,*s*)<*d*(*e*,*s*) or *d*(*e*_*i*_,*s*)=*d*(*e*,*s*) and *e*_*i*_≼*e*.

### Enumerating all edge-induced subgraphs

**Problem Definition:** Given an undirected graph *G*=(*V*,*E*), enumerate all connected edgesets, *CEIS*(*G*).

$$CEIS(G) = \{S \:|\: S \subseteq E \:and\: G[S]\: \text{is connected}\} $$

In this paper, we propose a reverse search algorithm for enumerating all connected edge sets of an undirected graph.

#### Search graph

For a single connected graph, the enumeration of the set of connected edgesets can be represented by a directed search graph in which nodes represent connected edgesets and there is a directed edge between two edgesets, (*X*,*Y*) if *Y*=*X*∪{*e*} and the deletion of *e* from *Y* keeps *X* connected. In the search graph, a search node (say, *Y*) can have multiple incoming edges as multiple connected edgesets can lead to the same connected edgeset. A naive approach to traverse the entire set of all connected edgesets is to grow an edgeset by extending it with one of its neighbor edges and checking in a global list whether the edgeset has been enumerated before to avoid duplicate listings. Given the combinatorial nature of connected edgesets, this approach is inefficient as it enumerates each edgeset many times, and the number of distinct edgesets grows exponentially with the size of the graph.

#### Reverse search

The algorithm builds and traverses the connected edgesets search tree wherein nodes in the tree correspond to connected edgesets and arcs correspond to the parent-children relations between these edgesets. The arcs in the search tree are defined by a neighborhood function that defines a set of search nodes that can be generated from a search node; this set is referred to as the valid children of a search node. The outgoing nodes of a search node constitute the valid search nodes that can be obtained from the search node. Each edgeset appears only once in the search tree, and there is only one incoming link to each edgeset from its unique parent edgeset. We enumerate the set of connected edgesets by depth-first traversal of the search tree. In this section, we define the *parent* operation and a data structure that allows for efficient *parent*/*child* operations.

#### Parent child relationship

If a search node *Y* corresponding to a connected edgeset can be obtained from a unique search node (say *X*), then *X* is called the parent node and *Y* is called the child node. The edgeset *X* can be obtained by deleting a specific edge from the edgeset *Y*.

##### **Lemma 1**

Let *U* be a connected edgeset with *s*=*anchor*(*U*) and *e*=*utmost*(*U*), then *G*[*U*−*e*] is also connected.

##### *Proof*

We will prove this claim by contradiction. Say, for a connected edgeset *U*, *e* is an edge with the longest shortest distance from *s* and for contradiction, assume that deleting *e* results in a disconnected graph. This means that there exists at least an edge *e*^′^ such that all shortest paths between *s* and *e*^′^ go through *e*. Let *p*_*ab*_ denote the shortest path between two edges *a* and *b* and *w*(*p*_*ab*_) denote the length of the path. Moreover, let $\phantom {\dot {i}\!}p_{se'} = \langle s,\cdots,e,\cdots,e' \rangle $ be a shortest path from *s* to *e*^′^ and $\phantom {\dot {i}\!}w(p_{se'})$ denote the length of the path. So, the shortest distance between *s* and *e*^′^, $\phantom {\dot {i}\!}w(p_{se}) + w(p_{ee'})$, is greater than the shortest distance between *s* and *e*, i.e., $\phantom {\dot {i}\!}w(p_{se'}) > w(p_{se})$. This contradicts our assumption that *e* is an edge with the longest shortest path distance from *s* in *U*. Thus, *G*[*U*−*e*] is connected. □

### Valid children

Building on Lemma 1, we can expand a node *U* in the search tree to construct one of its child nodes, *U*^∗^. For a connected edgeset *U* with *s*=*anchor*(*U*), *e*=*utmost*(*U*) and a neighboring edge *e*^′^∈*N*(*U*) such that *s*≼*e*^′^, the edgeset *U*^∗^=*U*∪{*e*^′^} is a valid child of *U* if and only if the following condition holds:

1. The distance from *s* to *e*^′^ is greater than the distance from *s* to *e*, or
2. Both *e*^′^ and *e* have the same distance to *s*, but *e*≼*e*^′^.

The definition of valid children ensures that the newly added edge to the child node has the longest distance from the anchor, and if multiple edges have the same longest distance to the anchor, the newly added edge is the largest. The proposed reverse search parent-child relation is the backbone of the enumeration tree neighborhood function, $\mathcal {N}: CEIS(G) \mapsto 2^{CEIS(G)}$. The parent-child relation guarantees that a search node only appears once in the range of the neighborhood function. We build a directed search graph whose nodes correspond to connected edgesets and there is a directed edge from node *X* to *Y* if edgeset *Y* is a child of *X*. For a child *Y*, let *X*=*P*(*Y*) denote its parent. The children of a connected edgeset *X*∈*CEIS*(*G*) in the search graph is defined as follows:

$$\mathcal{N}(X) = \{Y \in CEIS(G) \mid X = P(Y) \} $$

**Fig. 1:**
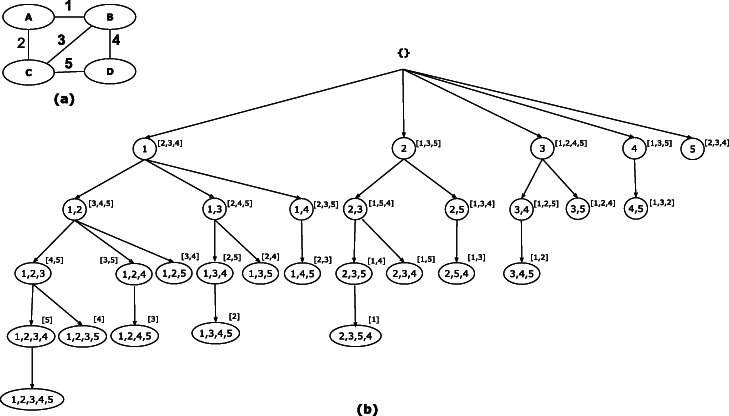
Enumeration tree for all connected edgesets

If *U* and *U*∪{*e*^′^} are edgesets corresponding to a parent and a child node, respectively, we call *e*^′^ a valid candidate of *U*, otherwise, we call it an invalid candidate of *U*. For an edgeset *U*, the set of neighboring edges, *N*(*U*), constitute the candidate edges and can be partitioned into valid and invalid candidates. Figure 1a shows a sample graph, and Fig. 1b shows the enumeration tree of the set of all connected edgesets of this graph. Edges are uniquely labeled starting from 1. The edges of an edgeset are written inside the oval shape and the set of candidate edges are written adjacent to the oval shape. Figure 1b shows that edgeset *U*={1,3} has {2,4,5} as the candidate edges; *anchor*(*U*)=1 and *utmost*(*U*)=3. Edge 2 is not a valid candidate because its distance to 1 is the same as the distance of the utmost edge 3 to edge 1 but 2 is less than 3 in the order of the edges, thus the branch corresponding to {1,3,2} will not be explored. Edge 4 has the same distance as edge 3 to edge 1, but since 4 is greater than 3, then edge 4 is a valid candidate. Edge 5 distance to edge 1 is larger than the distance of the utmost edge and thus it is a valid candidate. For the edgeset {2,5} with a candidate set {1,3,4}, both edges 1 and 3 are not valid candidates; edge 1 is less than the anchor 2 and edge 3 has the same distance as edge 5 to edge 2 but edge 3 is less than 5; edge 4 is a valid candidate because its distance to edge 2 is larger than the distance of the utmost edge 5 to edge 2. For single-edge search nodes in level 1, if the candidate edge is larger than the anchor edge, then it is an invalid edge.

### Enumerating all subgraphs of a single graph

Algorithm 1 shows the pseudo-code for our algorithm. For each edge in the graph, we call *EnumerateCEIS*, a recursive procedure. The procedure takes a connected edgeset *E*_*s*_, the set of candidate edges *C* and the utmost edge of the edgeset *utmost*(*E*_*s*_). For each edge *e*_*j*_ in the candidate set (line 6), the procedure checks if the edge is a valid candidate (line 7) for extending *E*_*s*_. If so, it updates the candidate set and recursively calls the *EnumerateCEIS* procedure (lines 8-9). The candidate set can be updated by using the current candidate set and the neighbors of the last added edge *N*(*e*_*j*_). To update the candidate set, we add the neighbors of the current edge *e*_*j*_ that are not already in the candidate set *C* or in the current edgeset *E*_*s*_ (line 10). The *isValidExtension* procedure (line 14) checks if the edge *e*_*j*_ is a valid candidate for the edgeset in *E*_*s*_ following the rules in the valid children section.

#### **Theorem 1**

Given an undirected graph *G*=(*V*,*E*), Algorithm 1 enumerates all connected edge-induced subgraphs without redundant enumeration.

#### *Proof*

Correctness means that each initial recursive call in Algorithm 1 (line 2) with *s* as the anchor edge will generate all the edge-induced subgraphs whose anchor is *s* under the enumeration tree rooted at *s*. First, all single edge subgraphs will be enumerated because we output the single edge the first time we call the recursive procedure (lines 2 and 5). We will prove that all connected edge-induced subgraphs with anchor *s* with at least two edges will be enumerated. For any connected edgeset *U*⊆*E*, *k*=|*U*|≥2 and *s*=*anchor*(*U*), we show this construction approach to obtain *G*[*U*]. Let *U*_*s*_ denote the sorted edges in *U*, *U*_*s*_={*e*_1_,*e*_2_,⋯,*e*_*k*_}, such that *e*_1_=*s* and for all 1≤*i*≤*k*−1 either *d*(*e*_*i*_,*s*)<*d*(*e*_*i*+1_,*s*) or *d*(*e*_*i*_,*s*) equals *d*(*e*_*i*+1_,*s*) and *e*_*i*_≼*e*_*i*+1_. There is a unique sequence of recursive calls to generate this *G*[*U*], starting the initial call with *E*_*s*_={*e*_1_} calling the procedure with *E*_*s*_={*e*_1_,*e*_2_} and ending the procedure with *E*_*s*_={*e*_1_,*e*_2_,⋯,*e*_*k*−1_} calling the last call with *E*_*s*_={*e*_1_,*e*_2_,⋯,*e*_*k*−1_,*e*_*k*_}. Each recursive call in this sequence will be executed because for all 2≤*i*≤*k*, the connected edgeset $E_{s}^{*}=\{e_{1},\cdots, e_{i}\}$ is a valid child of *E*_*s*_={*e*_1_,⋯,*e*_*i*−1_}. Note the last edge added in each call satisfies the valid child rules. This proves the completeness of the algorithm.

Next we show that the enumeration approach does not have redundant subgraph generation, i.e., each connected edge-induced subgraph is generated once. For a connected edge-induced subgraph, *G*[*U*], with *k*=|*U*| and *s*=*anchor*(*U*), let *U*_*s*_ be the set of sorted edges in *U* with respect to *s*, *U*_*s*_={*s*,*e*_2_,⋯,⋯,*e*_*k*_}. There is a unique path from the root *s* to the subgraph *G*[*U*]. The subgraph is obtained by starting from the subgraph *G*[{*s*}] and adding one edge at a time in the same order in *U*_*s*_. □


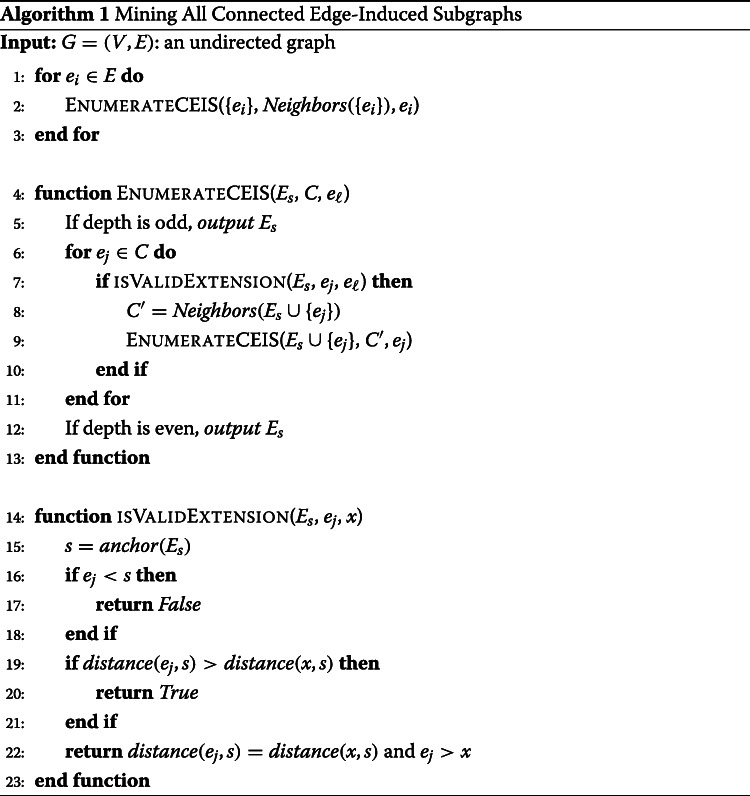


### Complexity analysis

Since the number of reported subgraphs can be exponential with respect to the number of edges of the graph, we analyze the time the algorithm takes to report the first subgraph and a subgraph after it has generated the previous subgraph [13]. This duration is defined as *Delay*. An enumeration algorithm is called a polynomial delay algorithm if its delay is polynomial in the input size [19]. The proposed connected edge-induced subgraph enumeration approach is a linear delay and this is an improvement of the current best cubic delay.

#### **Theorem 2**

Algorithm 1 is a linear delay and a linear space algorithm with respect to the number of edges of the graph.

#### *Proof*

We use an array-based implementation in which we maintain the set of edges of an edgeset, the candidate edges and the distance of the candidate edges to the anchor edge. Using this data structure, the anchor edge, utmost edges, and the distance of an edge to the anchor edge can be accessed in constant time. The algorithm checks if an edge is a valid candidate of the edgeset in a constant time *O*(1) (Algorithm 1 line 7). In the worst case scenario when all the candidate edges are invalid, the algorithm takes *O*(|*E*|) when the candidate set has all the edges. To prove the linear delay, we employ the alternative output method proposed in [19] to reduce the delay of the algorithm. The algorithm is an internal output algorithm since it outputs a solution for each recursive call. Following the alternative output method, the algorithm outputs a subgraph before starting to call the EnumerateCEIS recursive call if the depth of the recursive call is odd (Algorithm 1 line 5) and outputs the subgraph after the recursive calls for even depth (Algorithm 1 line 12). Therefore, each connected edgeset can be enumerated with linear delay *O*(|*E*|). For generating the first subgraph, the algorithm takes constant time since every initial recursive call (Algorithm 1 line 2) outputs a subgraph with single edge. If the graph has multiple connected components, we can run the algorithm for each component and the delay will be *O*(|*E*_*c*_|), where |*E*_*c*_| is the size of the largest connected component. □

An algorithm is *output polynomial*, if it outputs all the elements to be enumerated in time polynomial to the number of elements. Since the proposed algorithm takes linear time for each connected edgeset, it is output (or total) polynomial in the number of connected edgesets; output polynomial follows from the polynomial delay for each output. The algorithm explores the search tree in a depth first manner, which ensures that the space used is bounded by the depth of the search tree, which is at most |*E*|. We use three arrays, each of size |*E*| to keep track of which edges are in the connected edgeset, their neighbors, and their distances to the *anchor* edge. So, the depth first search of the enumeration tree can be done with linear space in the depth of the enumeration tree which is *O*(|*E*|).

## Mining frequent subgraphs

In many applications, we have a dataset of graphs and the goal is to extract significant subgraphs. In the frequent subgraph mining problem, the goal is to mine subgraphs that appear in at least a user-defined minimum threshold of the graphs. In this work, we are only concerned with connected frequent subgraphs.

**Graph Dataset**

**Fig. 2:**
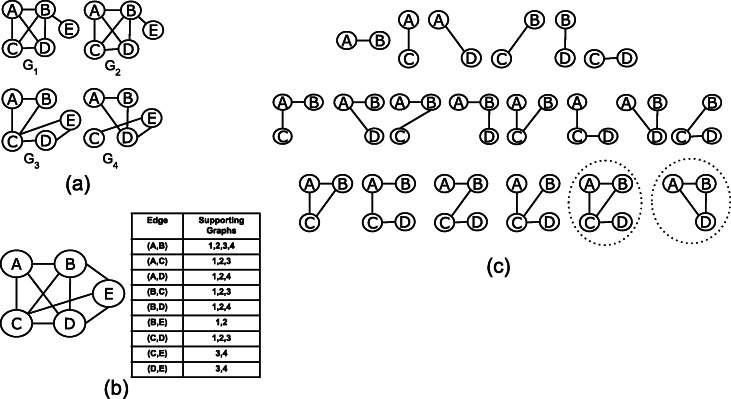
Mining Frequent Subgraphs: (**a**) Graph dataset (**b**) A summary graph representation and a table representing the edge attribute function *f* for mapping edges to the graphs in which they appear (**c**) Frequent and maximal frequent subgraphs with minimum support 3

Let $\mathcal {G} = \{G_{1}, G_{2}, \cdots, G_{n} \}$ denote a set of *n* undirected graphs. For an undirected graph *G*_*i*_=(*V*,*E*_*i*_), *i*∈[ [1⋯*n*] ], *V*={*v*_1_,*v*_2_,⋯,*v*_*k*_} denote the set of vertices and $E_{i} \subseteq \binom {V}{2}$ denote the set of edges of the corresponding graph. All the graphs are defined over the same set of vertices;

In this work, we represent the dataset ${\mathcal G}$ of *n* graphs as an edge-attributed graph, *G*=(*V*,*E*,*f*), where *V* is the set of vertices and *E* is the set of all the edges in the graph dataset and *f* is an edge attribute function. The edge attribute function *f* maps each edge to the set of graphs in which it appears. The set of all edges is the union of the sets of edges in each graph. We label the edges in the edge-attributed graph with unique identifiers {1,2,⋯,|*E*|}. Figure 2 shows a toy graph dataset of four graphs in (a) and the corresponding edge-attributed graph in (b).

**Supporting graphs**

Given a set of graphs $\mathcal {G}$, the set of supporting graphs of an edge-induced subgraph, *G*_*s*_ is defined as follow: $sup(\mathcal {G},G_{s})=\{G_{i}| G_{s} \subseteq G_{i} \; and \; G_{i} \in \mathcal {G}\}$. When the graph dataset is clear from the context, we refer to the supporting graphs as *sup*(*G*_*s*_). The cardinality of the supporting graphs is referred to as the support of the subgraph, i.e., |*sup*(*G*_*s*_)|.

**Frequent subgraph**

Given a graph dataset $\mathcal {G}$ and user-specified support threshold *S*_*min*_, a graph *G*_*s*_ is called frequent if the subgraph’s support is equals to or greater than the support threshold, i.e., *G*_*s*_ is a frequent subgraph if $|sup(\mathcal {G},G_{s})| \geq S_{min}$.

Since an edge-induced subgraph is uniquely identified by the edgeset, we use frequent subgraphs and frequent edgesets interchangeably.

**Problem definition**

Given a graph dataset $\mathcal {G}$ and a support threshold *S*_*min*_, the problem of mining the set of **frequent subgraph** is to enumerate the set:

$$\mathcal{F}=\left\{G_{s_{1}},G_{s_{2}},G_{s_{3}},\cdots,G_{s_{|{\mathcal F}|}}\right\} $$

such that every $G_{s_{i}} \in \mathcal {F}$ is a frequent connected subgraph, i.e., $|sup(\mathcal {G},G_{s_{i}})| \geq S_{min}$. For the graph dataset in Fig. 2a, the set of frequent subgraph for minimum support of 3 is shown in Fig. 2c. Given a minimum support threshold *S*_*min*_, the anti-monotone support constraint guarantees that if a subgraph *G*_*s*_ is frequent, then each subgraph *G*^∗^ of *G*_*s*_ is also frequent, i.e., |*sup*(*G*_*s*_)|≥*S*_*min*_ ⇒ for all *G*^∗^⊂*G*_*s*_, the subgraph is frequent |*sup*(*G*^∗^)|≥*S*_*min*_.


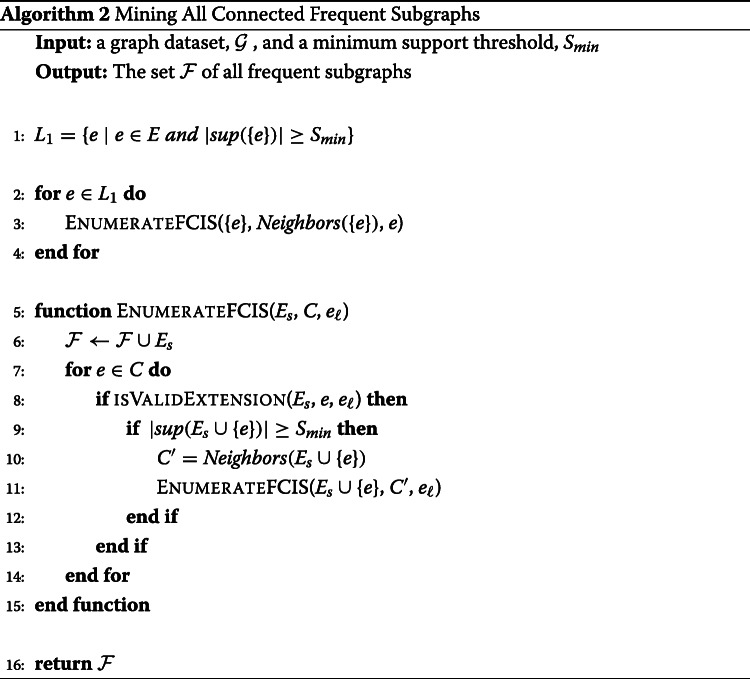


**Fig. 3:**
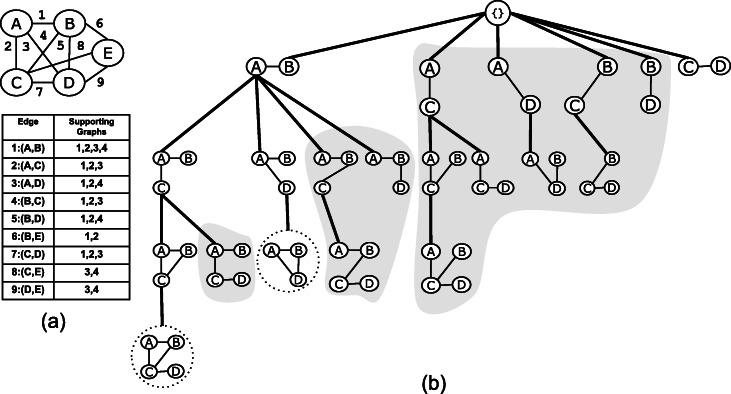
Mining Frequent and Maximal frequent Subgraphs: (**a**) A graph dataset of four graphs in the edge-attributed graph format (**b**) Enumeration tree for frequent subgraph with minimum support set to 3; maximal frequent subgraphs are circled with dotted line; all the subtrees with the light gray background are pruned when mining for only maximal frequent subgraphs in the proposed algorithm

Our proposed algorithm for mining all frequent subgraphs employs the reverse search enumeration approach in Algorithm 1 to enumerate all connected subgraphs and enforcing the supporting constraint. The anti-monotone property of the support of a subgraph is employed in the mining algorithm to prune search branches when an infrequent subgraph is encountered. If an infrequent subgraph is encountered, then the recursion procedure *EnumerateFCIS* is not called and the search subtree rooted at this infrequent subgraph is not enumerated. The enumeration tree for the set of frequent subgraphs is shown in Fig. 3b.

The algorithm for mining frequent subgraphs is shown in Algorithm 2. In line 1, infrequent edges are pruned, and the recursive *EnumerateFCIS* procedure is called for each frequent edge (Line 3). The recursive procedure follows the same steps as the enumeration approach in Algorithm 1, except for the *if* statement in line 9 to ensure that only search branches rooted at frequent subgraphs are explored. The recursive procedure is called only from frequent children (line 11). Therefore, only frequent subgraphs will be added to the set of frequent subgraphs in line 6.

### Mining maximal frequent subgraphs

Because of the downward closure property of frequent subgraphs where all the subgraphs of a frequent subgraph are frequent, there is high overlap between frequent subgraphs. A representative set of all frequent subgraphs is a concise summarization of the frequent subgraphs in the dataset. We thus propose an algorithm for mining maximal frequent subgraphs. Recall that a maximal frequent subgraph is a frequent subgraph that does not have any frequent supergraph. i.e., *G*[*E*_*s*_] is *maximal frequent* if there is no subgraph *G*[*E*^∗^]⊃*G*[*E*_*s*_], such that |*sup*(*G*[*E*^∗^])|≥*S*_*min*_. Though not efficient, all frequent subgraphs can be extracted from the set of maximal frequent subgraphs since all subgraphs of a maximal frequent subgraph are frequent. However, the exact frequency (support) of the frequent subgraphs can not be obtained from the maximal frequent subgraphs. Due to the combinatorial nature of frequent subgraphs, the set of maximal frequent subgraphs is much smaller than the set of all frequent subgraphs.

**Problem definition**

Given a graph dataset $\mathcal {G}$ and a support threshold *S*_*min*_, the problem of mining the set of **maximal frequent subgraph** is to enumerate the set:

$$\mathcal{M}=\{G_{s_{1}},G_{s_{2}},G_{s_{3}},\cdots,G_{s_{| \mathcal{M}|}}\} $$

such that every $G_{s_{i}} \in \mathcal {M}$ is a maximal frequent connected subgraph.

For the graph dataset of four graphs shown in Fig. 2a, and minimum support *S*_*min*_=3, there are two maximal frequent subgraphs and they are drawn inside dotted circles in Fig. 2c. These are the same subgraphs inside dotted circles in Fig. 3c.

In the enumeration tree for mining frequent subgraphs, every leaf search node is potentially a maximal frequent subgraph. The reason for a leaf not always being a maximal frequent subgraph is that there could be an invalid subgraph of that leaf that is frequent and it was not explored because it is not a valid extension at this stage of the enumeration tree. An algorithm for mining all maximal frequent subgraphs is to enumerate the frequent subgraphs enumeration tree and to report subgraphs that do not have any frequent valid or invalid extension. This algorithm is a straightforward extension of Algorithm 2. To decide locally if a subgraph is a maximal frequent subgraph, we need to switch lines 8 and 9 in Algorithm 2. We also need a flag before line 7 that is set to *true*. If the extended subgraph is frequent, |*sup*(*E*_*s*_∪{*e*})|≥*S*_*min*_, the flag is set to *false*, indicating that the subgraph is not maximal. After the for loop, we add the subgraph to the output list if the flag is still *true*. Note that this approach does not need a global list of the already mined maximal frequent subgraphs such as the one employed in the MULE algorithm. Following this mining approach, the enumeration tree for maximal frequent subgraphs would look like the tree in Fig. 3b. We will need to enumerate all 20 frequent subgraphs to get the two maximal subgraphs. Enumerating the search tree of frequent subgraphs is computationally expensive, especially for low minimum support thresholds when the search tree becomes very large. A more efficient approach would be to mine the set of all maximal frequent subgraphs without enumerating the whole frequent subgraphs enumeration tree. In the following subsections, we develop pruning strategies that eliminate the need to traverse search branches without sacrificing the correctness of the results. In the experiments section, we demonstrate how the proposed pruning strategies result in a significant performance improvement.

#### Consumed by a sibling

For a graph dataset $\mathcal {G}$, a connected frequent edgeset *S*⊆*E*, let *e*_*i*_ and *e*_*j*_ be two valid candidate edges of *G*[*S*] such that *e*_*i*_ is closer to *anchor*(*S*) than *e*_*j*_ and these two extensions generate two frequent subgraphs, *G*[*S*∪{*e*_*i*_}] and *G*[*S*∪{*e*_*j*_}] and the set of supporting graphs of *G*[*S*∪{*e*_*j*_}] is a subset of the supporting graphs of *G*[*S*∪{*e*_*i*_}], *sup*(*S*∪{*e*_*j*_})⊆*sup*(*S*∪{*e*_*i*_}). Under this scenario, these two subgraphs are not maximal frequent subgraphs because any maximal frequent subgraph that is a supergraph of *G*[*S*∪{*e*_*i*_}] will also be a supergraph of *G*[*S*∪{*e*_*j*_}]. This conclusion is reached by observing that any maximal subgraph that is a supergraph of *G*[*S*∪{*e*_*i*_}] can be extended with *e*_*j*_ without violating the minimum support threshold because we have the graphs that contain *G*[*S*∪{*e*_*i*_}] also contain *G*[*S*∪{*e*_*j*_}].

We will show that *G*[*S*∪{*e*_*i*_,*e*_*j*_}] is also a frequent subgraph that can be extended from *G*[*S*∪{*e*_*i*_}]. Note that since *sup*(*G*[*S*∪{*e*_*j*_}])⊆*sup*(*G*[*S*∪{*e*_*i*_}]), we get *sup*(*G*[*S*∪{*e*_*i*_,*e*_*j*_}])=*sup*(*G*[*S*∪{*e*_*j*_}]). Moreover, the maximal frequent subgraphs that are descendants of *G*[*S*] will be explored through the *G*[*S*∪{*e*_*i*_}] branch. In this case, we can safely prune the search branch rooted at *G*[*S*∪{*e*_*j*_}].

**Pruning rule 1: Consumed by sibling**For a connected frequent subgraph *G*[*S*] with two valid candidates *e*_*i*_≺_*S*_*e*_*j*_ resulting in two frequent children subgraphs *G*[*S*∪{*e*_*i*_}] and *G*[*S*∪{*e*_*j*_}] with *sup*(*G*[*S*∪{*e*_*j*_}])⊆*sup*(*G*[*S*∪{*e*_*i*_}]), the search branch rooted at *G*[*S*∪{*e*_*j*_}] can be safely pruned.

##### *Proof*

At the search node in the enumeration tree representing *G*[*S*], consider the set of edges *X*=*E*∖{*S*∪{*e*_*i*_,*e*_*j*_}} that can be reached from *S* and assume there is a maximal subgraph in the branch rooted at *G*:=*G*[*S*∪{*e*_*j*_}] which we plan to prune. Such a maximal frequent subgraph is denoted as *G*^′^=*G*[*S*∪{*e*_*j*_}∪*Y*], for *Y*⊆*X*. However, this *G*^′^ subgraph can be extended with *e*_*i*_ and still yield a frequent subgraph. The subgraph *G*[*S*∪{*e*_*j*_}∪*Y*∪{*e*_*i*_}] is a frequent subgraph since *e*_*i*_ is connected to *S* and can be added to *G*[*S*∪{*e*_*j*_}∪*Y*] without violating the minimum support threshold since *sup*(*G*[*S*∪{*e*_*j*_}])⊆*sup*(*G*[*S*∪{*e*_*i*_}]). The existence of a frequent subgraph *G*[*S*∪{*e*_*j*_}∪*Y*∪{*e*_*i*_}] contradicts our assumption that *G*[*S*∪{*e*_*j*_}∪*Y*] is a maximal frequent subgraph. This proves that *G*[*S*∪{*e*_*j*_}∪*Y*] is not a maximal frequent subgraph.

Moreover, *G*[*S*∪{*e*_*j*_}∪*Y*∪{*e*_*i*_}] is not a descendant of *G*[*S*∪{*e*_*j*_}] since *e*_*i*_ is not a valid extension to any descendant of *G*[*S*∪{*e*_*j*_}]. This is because once *e*_*j*_ is added to the edge set *S*, edge *e*_*i*_ can not be added since *e*_*i*_ is closer to *anchor*(*S*) than *e*_*j*_ and according to the valid children definition, *e*_*i*_ will never be a valid extension of any descendant of *G*[*S*∪{*e*_*j*_}]. Since all the subgraphs in the search tree rooted at *G*[*S*∪{*e*_*j*_}] can be expressed as *G*[*S*∪{*e*_*j*_}∪*Y*], for *Y*⊆*E*∖{*S*∪*e*_*i*_∪*e*_*j*_}, this proves that none of the descendants of *G*[*S*∪{*e*_*j*_}] will be a maximal frequent subgraph. Therefore, it is safe to prune the search branch rooted at *G*[*S*∪{*e*_*j*_}] without losing any maximal frequent subgraphs. □

For the graph dataset in Fig. 3, and minimum support *S*_*min*_=3, consider the edgeset {(*A*,*B*)} and its two neighboring edges (*A*,*C*) and (*B*,*C*). The supporting graphs of the subgraph induced by the edgeset {(*A*,*B*),(*A*,*C*)} are graphs {1,2,3} which is the same as the supporting graphs for the subgraph induced by the edgeset {(*A*,*B*),(*B*,*C*)}. Moreover, since edge (*A*,*C*) is closer than (*B*,*C*) to the anchor edge (*A*,*B*), then by applying Pruning Rule 1, the search branch at the subgraph induced by {(*A*,*B*),(*B*,*C*)} can be safely pruned. Similarly, the search branch at the subgraph induced by {(*A*,*B*),(*B*,*D*)} is pruned because it is subsumed by the search branch at {(*A*,*B*),(*A*,*D*)}.


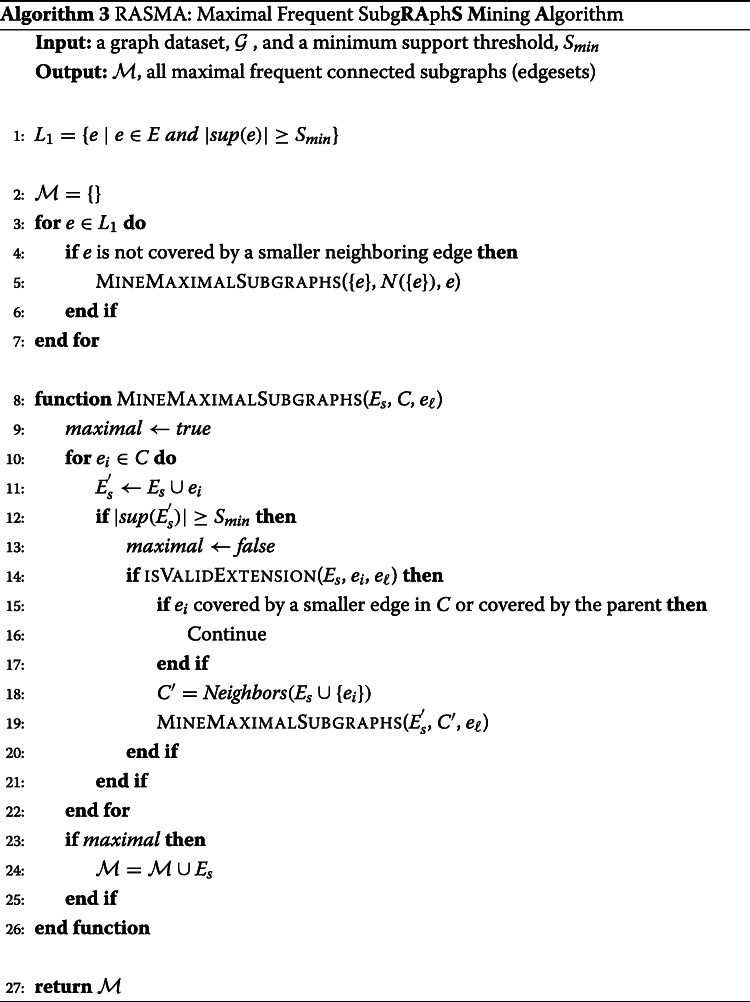


**Pruning rule 2: Identical as parent**For a graph dataset $\mathcal {G}$, a connected frequent edgeset *S*⊆*E* with two valid candidate edges *e*_*i*_≺_*S*_*e*_*j*_ that result in two frequent subgraphs such that the set of supporting graphs of *G*[*S*∪{*e*_*i*_}] is identical to the that of *G*[*S*]. Under this scenario, the entire search branch rooted at *G*[*S*∪{*e*_*j*_}] can be safely pruned.

For a given search node representing *G*[*S*], we sort all valid candidates based on their distances to the anchor edge of *S*, if we encounter an extension *e*_*i*_ such that the new extended subgraph appears in the same set of graphs as the parent subgraph, then all the remaining valid candidates in the sorted list of the parent can be safely skipped.

##### *Proof*

Assume that there is an edge *e*_*j*_ that is a valid extension of *G*[*S*] and *e*_*i*_ is closer to *anchor*(*S*) than *e*_*j*_. The supporting graphs of *G*[*S*∪{*e*_*j*_}] is a subset of the supporting graphs of *G*[*S*] (*sup*(*G*[*S*∪{*e*_*j*_}])⊆*sup*(*G*[*S*])) because the set of the supporting graphs of a subgraph is a subset of the set of the supporting graph of the parent subgraph. This pruning strategy is an extension of Pruning Rule 1. Since we have *sup*(*G*[*S*∪{*e*_*i*_}]) equals *sup*(*G*[*S*]) and therefore *sup*(*G*[*S*∪{*e*_*j*_}])⊆*sup*(*G*[*S*∪{*e*_*i*_}]), then by Pruning Rule 1, the search branch rooted at *e*_*j*_ can be safely pruned. □

For the example in Fig. 3, the supporting graphs of the subgraph induced by the edgeset {(*A*,*B*),(*A*,*C*),(*B*,*C*)} are the same as the supporting graphs of the parent subgraph {(*A*,*B*),(*A*,*C*)}, therefore all the remaining search branches for the parent graph can be safely pruned. This is the case for the subgraph induced by edgeset {(*A*,*B*),(*A*,*C*),(*C*,*D*)}.

##### Pruning rule 3: Level one pruning

Pruning for level one (single edge) is similar to pruning at any search node in the search tree. This Pruning Rule is the expansion of Pruning Rule 1 for the case of *S*=*∅*. For any two edges *e*_*i*_ and *e*_*j*_ sharing a common endpoint and *e*_*i*_ is smaller than *e*_*j*_, if the supporting graph of *e*_*j*_ is a subset of the supprting graphs of *e*_*i*_ (*sup*({*e*_*j*_})⊆*sup*({*e*_*i*_})), then the search tree rooted at *e*_*j*_ can be safely pruned. The proof follows the same steps as in Pruning Rule 1. For any connected edgeset *S*⊆*E*∖{*e*_*i*_,*e*_*j*_}, the subgraph *G*[{*e*_*j*_}∪*S*] is not a maximal frequent subgraph since *G*[{*e*_*j*_}∪*S*∪{*e*_*i*_}] is a frequent subgraph since *e*_*i*_ is connected to *e*_*j*_ and appears in all the graphs that *e*_*j*_ appears in. Moreover, *G*[{*e*_*j*_}∪*S*∪{*e*_*i*_}] is not a descendant of *G*[{*e*_*j*_}] since *e*_*i*_<*e*_*j*_.

In Fig. 3, the search subtree rooted at (*A*,*C*) is pruned because the set of supporting graphs of (*A*,*B*) is a superset of the supporting graphs of (*A*,*C*). Similarly the three search subtrees rooted at (*A*,*D*), (*B*,*C*), and (*B*,*D*) are all safely pruned by this rule.

### Algorithm

Algorithm 3 shows the proposed RASMA algorithm. The algorithm follows the enumeration approach for mining frequent subgraphs and employs the pruning strategies to avoid visiting subtree branches that will not result in maximal frequent subgraphs. In line 1, frequent edges are extracted and then in lines 3-7, a search subtree will be traversed from each frequent edge. Frequent edges that are covered by a neighboring smaller edge will not be explored by virtue of Pruning Rule 3 (line 4). In the MineMaximalSubgraph procuedure, for each edge in the candidate edges *C*, if the extension would generate a frequent subgraph, then we set the *maximal* flag to false indicating that the current subgraph is not a maximal frequent subgraph (lines 12-13). Next, for each valid candidate we check if this extension is covered by a previous extension (Pruning Rules 1 and 2: line 15) and we recursively call the procedure only for valid children that are not covered (lines 14-19). We add the current subgraph to the maximal frequent subgraphs set if the *maximal* flag is still true, line 24.

## Results

We tested the performance of RASMA on mining frequent and maximal frequent subgraphs from gene coexpression networks. Moreover, for investigating the impact of the pruning rules, we compared the running time of the algorithm with and without the pruning rules. All experiments were performed on a machine with Intel Xeon 2.40GHz processor with 16 Gbytes main memory, running the Linux operating system. The algorithms were implemented in C++ and the MULE implementation was in C.

### Performance on tissue gene expression

We tested the proposed algorithm on 35 tissue gene coexpression networks constructed by the Gene Genetic Network Analysis Tool [20]. The coexpression networks were inferred from Genotype-Tissue Expression (GTEx) data^1^. Each coexpression network is constructed from the gene expression of non-diseased tissue samples. On average there are 145,114 coexpression links (edges) in each network among 9,998 genes. In total, there are 1,548,622 unique coexpression edges that appear in at least one coexpression network. Among these edge, there are 55,558 edges that appear in at least 10 networks, 4,127 appear in at least 20 networks, and 554 appear in at least 30 networks. On average each edge appears in 3.28 networks.

Table 1 shows how the number of frequent and maximal frequent subgraphs ($|\mathcal {F}|$ and $|\mathcal {M}|$) and the running times for the MULE and RASMA for mining the frequent and maximal subgraphs vary for varying minimum support thresholds. For mining the maximal subgraphs, the proposed algorithm is orders of magnitude faster than the MULE algorithm for low support thresholds. The MULE algorithm is much slower for mining maximal frequent subgraphs since it has to enumerate the same frequent subgraphs enumeration tree. Moreover, for each potential maximal subgraph the MULE algorithm checks if it has a supergraph in a global list. For mining all the frequent subgraphs, both algorithms have similar running times and for a support threshold of 15 both did not finish the mining task in two days.

**Table 1:** Running times (in seconds) of the MULE and RASMA algorithms

sup	Frequent subgraphs			Maximal subgraphs		
*S*_*min*_	$|\mathcal {F}|$	MULE	Algorithm 2	$|\mathcal {M}|$	MULE	RASMA
20	214,119	0.93	**0.46**	4,701	5.25	**0.34**
19	599,798	1.02	**0.65**	6,463	12.32	**0.35**
18	1,776,157	2.01	**1.60**	9,038	50.75	**0.45**
17	9,040,719	7.79	**7.50**	12,879	241.19	**0.59**
16	2,968,677,772	2880	**1718**	18,978	1877.95	**0.92**
15	-	-	-	28,578	-	**3.50**

Table 2 shows the topological properties of the reported subgraphs and running times of RASMA for lower support thresholds. For calculating the topological properties of the maximal frequent subgraphs, only subgraphs with at least three edges (denoted $|\mathcal {M^{*}}|$) are considered since a large percentage of the maximal frequent subgraphs have one or two edges only. The number of maximal frequent subgraphs with at least three edges ($|\mathcal {M^{*}}|$) increases for lower support and so do the average numbers of edges ($\overline {|E|}$), nodes ($\overline {|V|}$), and density ($\overline {Density}$).

**Table 2:** Topological properties and running times for varying support thresholds

*S*_*min*_	$|\mathcal {M}|$	$|\mathcal {M^{*}}|$	$\overline {|E|}$	$\overline {|V|}$	$\overline {Density}$	Seconds
15	28,578	17,855	14.78	15.36	0.2	3.50
14	43,773	30,094	16.33	16.82	0.19	4.20
13	67,978	50,227	18.17	18.53	0.17	7.65
12	105,950	82,831	20.22	20.42	0.16	15.75
11	164,613	134,245	22.42	22.41	0.15	37.53
10	252,345	211,678	24.75	24.47	0.15	92.38
9	378,437	322,381	27.1	26.45	0.15	241.65
8	549,709	470,572	29.03	27.93	0.16	669.25
7	774,221	659,303	29.36	27.82	0.18	1958.77

#### Effectiveness of pruning rules

The pruning rules aim at reducing the number of search nodes explored while mining the maximal frequent subgraphs. The closer to the root the pruning occurs, the more search nodes are eliminated. To investigate how the proposed pruning rules improve the performance of the algorithm, we show a comparison with the pruning rules disabled. Table 3 shows the impact of the pruning rules on the running time and on the number of frequent subgraphs explored. Although both the frequent subgraphs and maximal subgraphs (without pruning) algorithms enumerate the same frequent subgraphs search tree, it is important to notice that the algorithm without pruning is much slower than mining all frequent subgraphs. This is true since for a maximal subgraph, all immediate potential children nodes have to be check for frequency to mark the subgraph as maximal (line 12 in Algorithm 3), regardless of whether the extension is a valid child. Therefore, the number of frequency checking is much larger than the number of frequent search nodes explored. However, the algorithm for frequent subgraphs checks if an extension is frequent only for the valid children (lines 8−9 in Algorithm 2). The algorithm with pruning strategies traverses only a very small fraction (0.000028 for support = 16) of the frequent subgraphs while maintaining the completeness of the maximal frequent subgraphs.

**Table 3:** Impact of the pruning strategies on the number of search nodes explored

sup	maximal	Without Pruning		RASMA	
*S*_*min*_	$|\mathcal {M}|$	#Nodes	Seconds	#Nodes	Seconds
20	4,701	214,119	0.35	13,869	**0.34**
19	6,463	599,798	2.54	20,529	**0.35**
18	9,038	1,776,157	8.18	31,442	**0.45**
17	12,879	9,040,719	51.46	49,880	**0.59**
16	18,978	2,968,677,772	21,031.44	82,607	**0.92**

#### Analysis of maximal frequent subgraphs

We performed a biological enrichment for the gene sets (nodes) of the maximal frequent subgraphs. A biological annotation, knowledge, is to said to be enriched in a gene set if a significant subset of the genes of the gene set are annotated with the given annotation. We tested the overrepresentation of genes with a specific annotation in a gene set using the hybergeometric test (with *pvalue*=0.01). We used multiple annotation databases from the Molecular Signatures Database (MSigDB) [21, 22] for assessing the enrichment of the genes in these reported subgraphs with these annotations.

1. KEGG: Gene sets derived from the KEGG pathway database (186 sets).
2. BP: Gene sets derived from the GO Biological Process Ontology. (7350 sets).
3. MF: Gene sets derived from the GO Molecular Function Ontology. (1645 sets).

Table 4 shows the percentage of the maximal frequent subgraphs whose genesets are biologically enriched for each of the three biological signatures. Some subgraphs are enriched with several signatures and some signatures are enriched in the genes of multiple subgraphs. Moreover, the reported subgraphs are enriched with a large number of biological annotations for each of the databases. Only maximal frequent subgraphs with at least three edges were considered for the analysis (denoted as $\mathcal {|M^{*}|}$ in the table). The enrichment analysis shows that frequent subgraphs are highly enriched with known biological annotations.

**Table 4:** Enrichment analysis of the maximal frequent subgraphs

sup	maximal*	Topological Properties		Enrichment Analysis		
*S*_*min*_	$|\mathcal {M^{*}}|$	$\overline {|E|}$	$\overline {Density}$	*E*_*KEGG*_	*E*_*BP*_	*E*_*MF*_
20	1,701	9.82	0.29	76.3	91.5	86.5
19	2,580	10.76	0.26	77.2	91.9	87.1
18	4,116	11.68	0.25	77.8	91.8	87.5
17	6,598	12.72	0.23	79.2	91.7	87.7
16	10,716	14.04	0.21	81.0	91.8	88.7

An annotation can be enriched in many reported gene sets. We sorted the annotations by the number of subgraphs they are enriched in. Table 5 shows the top biological signatures that were enriched the most in the reported genesets of the maximal frequent subgraphs for *S*_*min*_=20.

**Table 5:** Top enriched signatures in maximal subgraphs; *S*_*min*_=20

KEGG Pathways	N	KEGG Pathways	N
RIBOSOME	884	HUNTINGTONS_DISEASE	274
PARKINSONS_DISEASE	261	OXIDATIVE_PHOSPHORYLATION	257
ALZHEIMERS_DISEASE	251	CARDIAC_MUSCLE_CONTRACTION	158
VIRAL_MYOCARDITIS	21	TIGHT_JUNCTION	18
AUTOIMMUNE_THYROID_DISEASE	17	SPLICEOSOME	16
**GO Biological Process**	N	**GO Molecular Function**	N
VIRAL_GENE_EXPRESSION	888	RRNA_BINDING	511
RNA_CATABOLIC_PROCESS	886	ELECTRON_TRANSFER_ACTIVITY	223
TRANSLATIONAL_INITIATION	884	MRNA_BINDING	205
PROTEIN_TARGETING	884	NADH_DEHYDROGENASE_ACTIVITY	133
CYTOPLASMIC_TRANSLATION	808	ANTIGEN_BINDING	113
RIBOSOME_BIOGENESIS	777	5S_RRNA_BINDING	69
RIBOSOME_ASSEMBLY	722	CADHERIN_BINDING	39

### Frequent coexpression subnetworks for breast cancer stages

We constructed gene coexpression networks from breast cancer gene expression samples in the TCGA portal. We downloaded 1,310 RNA-seq samples; 113 of these samples are Solid Tissue Normal (used as control) and the remaining 1,197 samples are Primary Solid Tumor. The cancer samples belong to four different stages. For cancer and control samples for each stage, we extracted the differentially expressed genes (DEGs) using |*log*(*FC*)|>2 and corrected *p*- *value*<0.05 as the cutoffs. We then constructed the coexpression network among the DEGs for each cancer stage. A pair of differentially expressed genes is considered coexpressed if absolute value of the Pearson correlation coefficient (PCC) is at least 0.7. Table 6 summarizes the number of samples, DEGs, and coexpression links for each cancer stage. There are 1,176 common DEGs genes that are dysregulated in all the stages and 2,394 unique DEGs in all the stages. Moreover, there are 9,677 common links that appear in all the four coexpression networks, and 81,204 unique links in all the networks. We mined the maximal frequent subnetworks for support thresholds of 2, 3 and 4. Table 7 show the topological and biological enrichment analysis of the reported subnetworks. For the biological enrichment analysis, we used the following biological collections from the Molecular Signatures Database.

1. KEGG: Gene sets derived from the KEGG pathway database (186 sets).

**Table 6:** Cancer stages

Stage	samples	DEGs	coexpression links
I	203	1,656	25,368
II	691	1,694	19,230
III	281	1,700	21,697
IV	22	1,903	68,905

**Table 7:** Topological and enrichment analysis of the maximal frequent subgraphs

sup	Maximal	size ≥3	Topological Properties			Enrichment Analysis			
*S*_*min*_	$|\mathcal {M^{}}|$	$|\mathcal {M^{*}}|$	$\overline {|E|}$	$\overline {|V|}$	$\overline {Density}$	*E*_*KEGG*_	*E*_*CGN*_	*E*_*CM*_	*E*_*OS*_
2	143	68	1334.62	48.85	0.5	0.53	0.29	0.5	0.37
3	69	31	1503.06	57.97	0.51	0.65	0.29	0.48	0.29
4	32	10	965.5	41.4	0.58	0.5	0.2	0.4	0.3
2. CGN: computational cancer gene neighborhoods (427 sets).
3. CM: computational cancer modules collected from a variety of resources (431 sets).
4. OS: Oncogenic signatures of cellular pathways which are often dis-regulated in cancer (189 sets).

Table 8 shows the top enriched oncogenic signatures in the gene sets of the maximal frequent subnetworks for varying support thresholds. Note that the number of enriched signatures can be different than the number of enriched subnetworks as a gene set in a subnetwork can be enriched with several signatures. The table also shows the number of subnetworks in which each signature is enriched. For *S*_*min*_=2, the most enriched signature is RB_P130_DN.V1_UP. This oncogenic signature represents up-regulated genes in primary keratinocytes from RB1 and RBL2 [23]. The RB1 gene has a role in proliferation and apoptosis and the alteration of RB1 underlies both cancer development and resistance to therapy [24]. Mutational loss of RB1 has been linked to the development of breast cancer [25]. There are three oncogenic signatures that are highly enriched in the gene sets of the subnetworks for all the support thresholds.

1. E2F1_UP.V1_UP: Genes up-regulated in mouse fibroblasts over-expressing E2F1 [26].

**Table 8:** Top enriched oncogenic signatures

*S*_*min*_	Signatures	# subnetworks
2	RB_P130_DN.V1_UP	7
	CORDENONSI_YAP_CONSERVED_SIGNATURE	7
	E2F1_UP.V1_UP	6
	EGFR_UP.V1_DN	6
	ERBB2_UP.V1_DN	6
3	E2F1_UP.V1_UP	4
	EGFR_UP.V1_DN	4
	ERBB2_UP.V1_DN	4
	GCNP_SHH_UP_EARLY.V1_UP	4
4	E2F1_UP.V1_UP	1
	EGFR_UP.V1_DN	1
	ERBB2_UP.V1_DN	1
	GCNP_SHH_UP_EARLY.V1_UP	1
2. EGFR_UP.V1_DN: Genes down-regulated in MCF-7 cells (breast cancer) positive for ESR1 and engineered to express ligand-activatable EGFR [27].
3. ERBB2_UP.V1_DN: Genes down-regulated in MCF-7 cells (breast cancer) positive for ESR1 and engineered to express ligand-activatable ERBB2 [27].

## Conclusion

Frequent coexpression subnetworks have been shown to be effective in functional annotation and subnetwork biomarker discovery. We proposed a reverse search algorithm for mining maximal frequent subgraphs. We first proposed a reverse search strategy for enumerating all edge-induced subgraphs from a single graph. The enumeration approach is then employed for mining frequent and maximal subgraphs. To eliminate search branches that will not result in maximal frequent subgraphs, we proposed pruning strategies that employ the order in which branches are enumerated. The pruning strategies are possible because the reverse search enforces strict definition on the order in which search nodes are enumerated. Experiments on gene coexpression datasets demonstrate the effectiveness of the proposed approaches. The proposed approach is thousands of times faster than the existing algorithm. Enrichment analysis of the genesets in the maximal frequent subgraphs reveal that maximal frequent coexpression subnetworks are enriched with known biological annotations.

## Data Availability

The dataset and the implementation of the algorithm is available at http://www.cs.ndsu.nodak.edu/~ssalem/software/rasma.html.

## Footnotes

1 https://www.gtexportal.org/
